# Correlation enhanced distribution adaptation for prediction of fall risk

**DOI:** 10.1038/s41598-024-54053-5

**Published:** 2024-02-12

**Authors:** Ziqi Guo, Teresa Wu, Thurmon E. Lockhart, Rahul Soangra, Hyunsoo Yoon

**Affiliations:** 1 Department of Systems Science and Industrial Engineering, The State University of New York at Binghamton, Binghamton, USA; 2 School of Computing and Augmented Intelligence, Arizona State University, Tempe, USA; 3 School of Biological and Health Systems Engineering, Arizona State University, Tempe, USA; 4 Department of Physical Therapy, Chapman University, Orange, USA; 5 Department of Industrial Engineering, Yonsei University, Seoul, Korea

**Keywords:** Unsupervised domain adaptation, Machine learning, Classification, Fall risk, Biomedical engineering, Classification and taxonomy, Data integration, Computational biology and bioinformatics, Health care, Engineering

## Abstract

With technological advancements in diagnostic imaging, smart sensing, and wearables, a multitude of heterogeneous sources or modalities are available to proactively monitor the health of the elderly. Due to the increasing risks of falls among older adults, an early diagnosis tool is crucial to prevent future falls. However, during the early stage of diagnosis, there is often limited or no labeled data (expert-confirmed diagnostic information) available in the target domain (new cohort) to determine the proper treatment for older adults. Instead, there are multiple related but non-identical domain data with labels from the existing cohort or different institutions. Integrating different data sources with labeled and unlabeled samples to predict a patient's condition poses a significant challenge. Traditional machine learning models assume that data for new patients follow a similar distribution. If the data does not satisfy this assumption, the trained models do not achieve the expected accuracy, leading to potential misdiagnosing risks. To address this issue, we utilize domain adaptation (DA) techniques, which employ labeled data from one or more related source domains. These DA techniques promise to tackle discrepancies in multiple data sources and achieve a robust diagnosis for new patients. In our research, we have developed an unsupervised DA model to align two domains by creating a domain-invariant feature representation. Subsequently, we have built a robust fall-risk prediction model based on these new feature representations. The results from simulation studies and real-world applications demonstrate that our proposed approach outperforms existing models.

## Introduction

Vast volumes of unlabeled data are generated and made available in numerous domains. In the context of machine learning, a domain refers to a subset of the larger data space that is relevant for a specific task or application. However, acquiring sufficient labeled data can be exceedingly costly and sometimes impractical. For example, on average, each pixel-level image in the Cityscapes dataset required 1.5 h to complete the annotation^1^. Domain adaptation (DA) addresses the limited labeled data issue by aligning two distinct datasets: one from a source domain and the other from a target domain. The source domain contains a large amount of labeled data on which classifiers can be reliably trained. The target domain broadly refers to a dataset assumed to have different characteristics from the source domain, where those classifiers are applied. Several example scenarios require domain adaptation (DA). In computer vision tasks, objects might come from multiple sources, each with different backgrounds, object styles, and locations^2–6^. In activity recognition tasks, sensors might be placed in different body locations^7,8^. In speech recognition, voices may come from different speakers^9,10^. In sentiment analysis, various text sources, such as electronics or DVDs, are used for analysis^11,12^. In healthcare, acquiring labeled data and large samples is even more challenging. For instance, in medical image analysis, the major challenge in constructing reliable and robust models is the lack of labeled data^13,14^. Clinical outcomes might be sourced from different machines and healthcare providers. Variations between different data sources can significantly reduce prediction accuracy. These problems are studied in DA, where the model is learned on one dataset (i.e., source domain) and then transferred to a target dataset (i.e., target domain) with different distribution properties.

Although machine learning approaches for supervised learning have performed well, they assume that training and testing data are drawn from the same distribution, which may not always be true. To complement this challenge, DA aims to align the target to the source domain by creating a domain-invariant feature representation. After adaptation, it becomes a standard machine learning problem that assumes test data are drawn from a similar distribution as the training data. In this paper, we propose an unsupervised DA method that specifically addresses situations where labeled data are available only in the source domain, and the target domain is unlabeled, which is common in practice.

According to a literature review^15^, existing DA methods can be organized into two categories: (a) feature transformation and (b) instance weighting. Feature transformation either performs feature space alignment by exploring the subspace geometrical structure, such as subspace alignment (SA)^16^, CORrelation ALignment (CORAL)^17^, and geodesic flow kernel (GFK)^5^, or distribution adaptation to reduce the distribution divergence between domains, such as transfer component analysis (TCA)^18^ and joint distribution adaptation (JDA)^19^. Instance reweighting reweights the samples from the source domain to the target based on the weighting methods^20,21^. The challenge with existing methods is degenerated feature transformation^22^, where both subspace alignment and distribution adaptation can reduce the divergence between domains but not eliminate it. Subspace alignment only considers the subspace or manifold structure, failing to achieve complete feature alignment. Conversely, distribution adaptation reduces the distribution distance in the original feature space but often distorts features, making it more challenging to reduce the divergence. Therefore, exploiting both the advantages of subspace alignment and distribution adaptation is significant for further developing DA. This study proposes a novel DA method to address this challenge.

Unsupervised learning assumes the availability of labeled source data and unlabeled target data. Several unsupervised domain adaptation (DA) methods are described in a literature review^23^. Domain-invariant feature learning methods aim to align the source and target domains by creating a domain-invariant feature representation, where features follow the same distribution regardless of the input's source or target domain. Typically, this is achieved through a feature extractor neural network^17,24–26^. Domain mapping methods, on the other hand, use adversarial techniques to create a pixel-level map from one domain to another, often accomplished with a conditional GAN^27–29^. Normalization statistics methods leverage normalization layers like batch normalization commonly found in neural networks^30,31^. Existing unsupervised DA methods predominantly emphasize neural network-based approaches, but they may perform poorly in cases with a small sample size and a limited number of features. This can be attributed to the fact that neural networks typically require large amounts of data to learn meaningful representations and can suffer from overfitting when the number of features is limited. Therefore, to address this shortcoming, we propose our shallow unsupervised DA approach, Correlation Enhanced Distribution Adaptation (CEDA).

Domain adaptation has garnered considerable attention in healthcare applications in recent years, particularly in computer-aided medical image analysis^32–34^, due to its ability to reuse pre-trained models from related domains. Many other healthcare problems also face the challenge of lacking labeled data. This study extends the application of domain adaptation, especially unsupervised DA, to sensor-based prognosis.

Of particular interest in this research is fall detection. Falls pose significant threats to the health of older adults and can hinder their ability to remain independent. As CDC reports suggest, 3 million older people are treated in emergency departments for fall injuries each year, and fall death rates in the U.S. increased by 30% from 2007 to 2016. Therefore, fall prevention is a critical component of healthcare for the senior community. In the realm of fall risk assessment, particularly for older adults, there is a recognized importance of both intrinsic and extrinsic factors. Intrinsic factors include muscle strength^35^, balance^36^, and gait stability^37^, whereas extrinsic factors involve elements like home hazards and footwear choices^38^. Recently, wearable sensors have become invaluable in assessing fall risk, especially through the use of accelerometers and gyroscopes to capture a variety of movement characteristics. Diverse feature sets have been explored in fall risk assessment, including nonlinear dynamics. Measures such as Shannon entropy and frequency analysis, which reflect gait dynamics, have shown significantly higher values in individuals prone to falls, indicating their potential as fall risk predictors^39^. Nonlinear metrics, like multiscale entropy (MSE) and recurrence quantification analysis (RQA) applied to trunk accelerations, have demonstrated positive correlations with fall histories, suggesting their utility in identifying individuals at higher risk^40^. Koshmak et al. employed supervised feature learning to estimate fall risk probabilities, underscoring the critical importance of feature selection in effective assessment^41^. Additionally, research has highlighted the significance of integrating gait and posture analysis for enhanced precision in predicting fall risks^42^. Recent studies collectively emphasize the substantial potential of wearable sensors in delineating fall risk, particularly through examining features like entropy, complexity, multiscale entropy, and fractal properties^43–45^.

This study proposes a novel approach for fall prediction using the 10-m walking test. We focus on the challenge where the fall information for the target group is unknown, while it is known for the other group. As they are different groups of people, their characteristic distributions (marginal and conditional) differ. Hence, directly using data from one group to train the classification models would not provide accurate predictions for the other group.

## Methods

### Formulation

Without loss of generality, we describe our method by taking a binary classification problem as the running example. The proposed formula can be directly applicable to multi-class classification problems. Assume source-domain training examples ${D}_{S}=\left\{\overrightarrow{{x}_{i}}\right\}$, $\overrightarrow{x}\in {\mathbb{R}}^{D}$ with labels ${L}_{s}=\{{y}_{i}\}$, $y\in \left\{1,,\dots ,L\right\},$ and target data ${D}_{T}=\left\{\overrightarrow{{u}_{i}}\right\}, \overrightarrow{u}\in {\mathbb{R}}^{d}$. Both $\overrightarrow{x}$ and $\overrightarrow{u}$ are the d-dimensional feature representations $\phi \left(I\right)$ of input $I$.

### Proposed method

We propose the Correlation Enhanced Distribution Adaptation (CEDA) model, which combines and improves upon the CORrelation ALignment (CORAL) and Joint Distribution Adaptation (JDA) approaches, outperforming each of these methods individually. In the following section, we will provide a brief introduction to these two approaches: CORrelation ALignment (CORAL) and Joint Distribution Adaptation (JDA).

1. CORrelation ALignment (CORAL)^17^ transforms the source features to the target space by aligning the second-order statistic, the covariance. The covariances differ in the original source and target domain distributions. The researchers propose conducting source decorrelation to remove the feature correlation of the source domain and then constructing target re-correlation by adding the correlation of target features to the source domain. After these two steps, the two distributions are well aligned, and the classifiers trained on the adjusted source domain work well in the target. However, this method aligns the source distributions as a whole to the target domain, neglecting the significance of individual samples.
2. Joint Distribution Adaptation (JDA)^19^ aims to find a feature transformation that jointly minimizes the difference in marginal and conditional distributions between domains. Although no labeled data exists in the target domain, this method generates pseudo-target labels by applying a classifier ƒ trained on the adapted labeled source to the unlabeled target. Iterative label refinement is used to improve the classifier and labeling quality. However, it has limitations in generating accurate pseudo labels for the target domain."

Our proposed method begins by employing CORAL as the first step for source decorrelation, which involves removing the feature correlation of the source domain and adding the correlation of the target to the source domain. This integrated adaptation aims to roughly align the source samples to the target domain. However, due to the presence of distribution noise, some samples may not be correctly aligned, leading to suboptimal results. To ensure accurate alignment for all samples, a further meticulous adaptation is performed. In the second step of our proposed method, we apply Joint Distribution Adaptation (JDA) to the adjusted source samples obtained from the first step. JDA has a limitation of generating pseudo-target labels in the first iteration, which can result in an inappropriate adjustment in the conditional distribution. To overcome this challenge, we utilize CORAL to provide an initial adjusted source sample for JDA. The transformed target samples are then classified using a 1-Nearest Neighbor (1NN) classifier, trained with the transformed new source samples.

Moreover, CORAL serves as a nonparametric model that does not require any parameter tuning, making it highly advantageous for unsupervised learning. It aligns the distribution of source and target features in an unsupervised manner. In our approach, CORAL transforms the source feature ${\mathbf{X}}_{S}$ to the target space ${\mathbf{X}}_{{\text{T}}}$ by aligning the second-order statistic, the covariance. After obtaining new ${\mathbf{X}}_{S}$ by multiplying the CORAL adaptation matrix (A_CORAL) with ${\mathbf{X}}_{S}$, we train a standard classifier ƒ (nearest neighbor in our case) on the new ${\mathbf{X}}_{S}$ to generate the initial pseudo-target labels ${\widehat{{\varvec{y}}}}_{T}$ for the target. Subsequently, we build an MMD (Maximum Mean Discrepancy) matrix $\mathbf{M}$ (Gretton et al., 2008):

1 $$\begin{array}{c}{\left({M}_{0}\right)}_{ij}=\left\{\begin{array}{c}\frac{1}{{n}_{s}{n}_{s}}, \quad{x}_{i}, {x}_{j}\in {\mathcal{D}}_{s}\\ \frac{1}{{n}_{t}{n}_{t}}, \quad{x}_{i}, {x}_{j}\in {\mathcal{D}}_{t}\\ \frac{-1}{{n}_{s}{n}_{t}}, \quad otherwise\end{array}\right.\end{array}$$

which is adopted as the distance measurement for the objective of reducing the difference between marginal distributions ${P}_{s}\left({{\varvec{X}}}_{s}\right)$ and ${P}_{t}({{\varvec{X}}}_{T}$). An MMD matrix ${\left\{{\mathbf{M}}_{C}\right\}}_{c=1}^{C}$ is then constructed based on class labels, used as the distance measurement for minimizing the difference between conditional distribution, as follows:

2 $$\begin{array}{c}{\left({M}_{c}\right)}_{ij}=\left\{\begin{array}{c}\frac{1}{{n}_{s}^{\left(c\right)}{n}_{s}^{\left(c\right)}}, {x}_{i}, {x}_{j}\in {\mathcal{D}}_{s}^{\left(c\right)}\\ \frac{1}{{n}_{t}^{\left(c\right)}{n}_{t}^{\left(c\right)}}, {x}_{i}, {x}_{j}\in {\mathcal{D}}_{t}^{\left(c\right)}\\ \\ \frac{-1}{{n}_{s}^{\left(c\right)}{n}_{t}^{\left(c\right)}}, \left\{\begin{array}{c}{x}_{i}\in {\mathcal{D}}_{s}^{\left(c\right)}, {x}_{j}\in {\mathcal{D}}_{t}^{\left(c\right)}\\ {x}_{j}\in {\mathcal{D}}_{s}^{\left(c\right)}, {x}_{i}\in {\mathcal{D}}_{t}^{\left(c\right)}\end{array}\right.\\ 0, otherwise\\ \end{array}\right.\end{array} $$

Next, the optimal adaptation matrix A is calculated by solving Eq. (3) for the k smallest eigenvectors, and ${\varvec{Z}}:={{\varvec{A}}}^{T}{\varvec{X}}$:

3 $$(\mathbf{X}{\sum }_{{\text{c}}=0}^{{\text{C}}}{\mathbf{M}}_{{\text{c}}}{\mathbf{X}}^{{\text{T}}}+\uplambda \mathbf{I}) \mathbf{A}=\mathbf{X}\mathbf{H}{\mathbf{X}}^{{\text{T}}}\mathbf{A}{\varvec{\Phi}}$$

A standard classifier $\fancyscript{f}$ is trained on $({\mathbf{A}}_{{\text{S}}}^{{\text{T}}}{\mathbf{X}}_{{\text{s}}}, {{\varvec{y}}}_{S})$ to generate ${\widehat{{\varvec{y}}}}_{T}:=\fancyscript{f} ({\mathbf{A}}_{T}^{T}{{\varvec{X}}}_{T})$. If we use this labeling ${\widehat{{\varvec{y}}}}_{T}$ as the pseudo-target labels and run JDA iteratively, we can alternate improving the labeling quality until convergence. The model will return adaptation matrix $\mathbf{A}$, embedding $\mathbf{Z}$, adaptive classifier *ƒ,* with the input of source data ${\mathbf{X}}_{{\text{S}}}$, ${\mathbf{y}}_{{\text{s}}}$, target Data ${\mathbf{X}}_{{\text{T}}}$; #subspace bases $k$, regularization parameter $\uplambda$.

The algorithm is summarized in the following pseudo-code:

**Algorithm:**
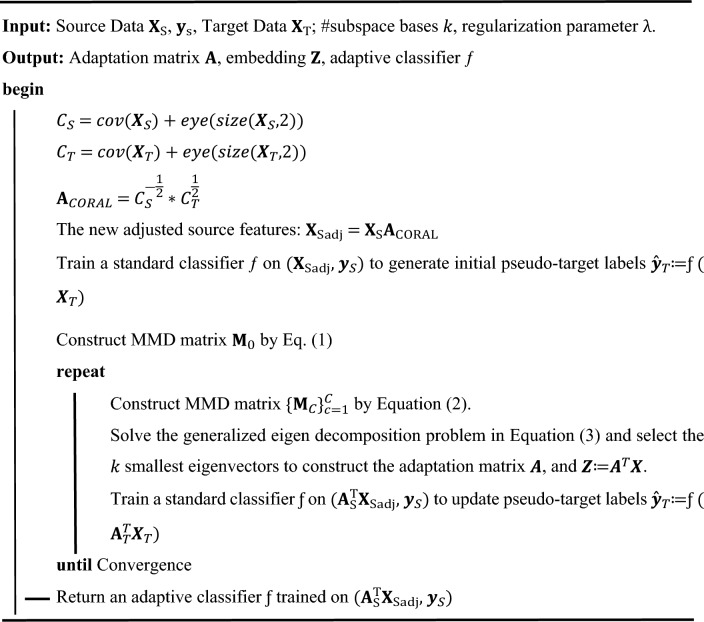
CEDA for unsupervised DA.

## Simulation study

This section uses simulation data to demonstrate the proposed method’s performance under several scenarios. The simulation data are generated as follows: the source and target domain data are sampled from a multi-dimensional normal distribution with randomly selected parameter setting. We consider a binary classification. In the source domain, the simulation data ${{\varvec{X}}}_{{\varvec{s}}}\sim \mathcal{N}({{\varvec{\mu}}}_{{\varvec{s}}},\boldsymbol{ }{{\varvec{\Sigma}}}_{\mathbf{s}})$ with corresponding responses ${{\varvec{Y}}}_{{\varvec{s}}}\in \{\mathrm{0,1}\}$ and ${{\varvec{X}}}_{{\varvec{t}}}\sim \mathcal{N}\left({{\varvec{\mu}}}_{{\varvec{t}}},\boldsymbol{ }{{\varvec{\Sigma}}}_{\mathbf{t}}\right),{{\varvec{Y}}}_{{\varvec{t}}}\in \{\mathrm{0,1}\}$ for the target domain.

### Impact of sample size on model performance

**Figure 1:**
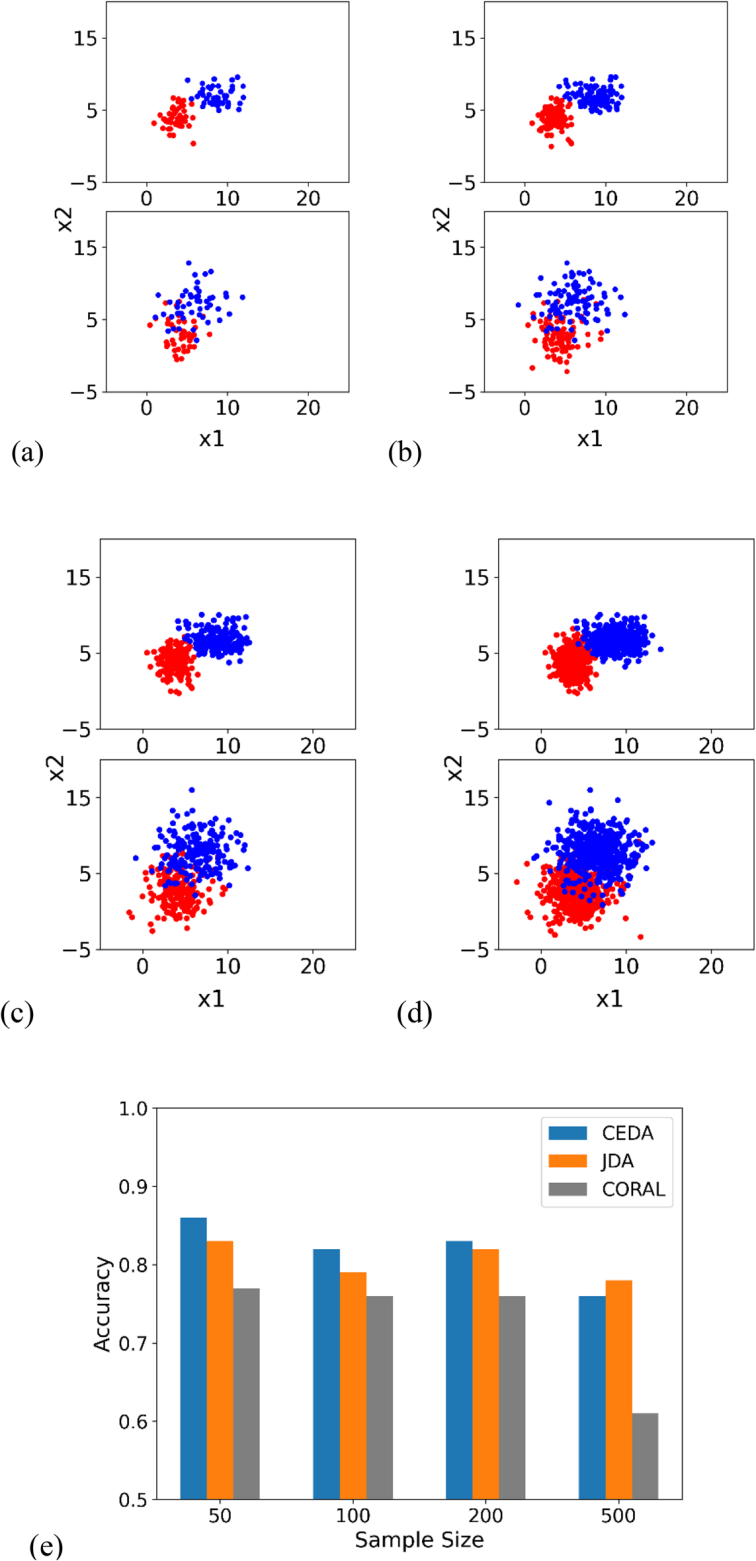
Scatter plots of source samples (in upper plots) and target samples (in lower plots). We visualize the first and second dimension. Two colors (red and blue) represent two classes. (**a–d**) Have 50, 100, 200, and 500 samples, respectively. (**e**) Denotes the classification accuracies at different sample sizes.

In the simulation setup, while maintaining the sample mean and covariance values, change the number of samples in each class. Each dataset is constructed by randomly selecting parameter values within predefined ranges. Specifically, the mean vector μ is randomly drawn from a uniform distribution within the interval^2,5^ for red class and^4,9^ for blue class, across each dimension. Similarly, the covariance matrix Σ is generated by first randomly selecting diagonal elements from a uniform distribution within the range^1,3^ for source samples^4,6^, for target, and then applying a random orthogonal transformation to introduce off-diagonal covariance components. The dimension for each class is the same and is randomly selected from a uniform distribution within the interval^2,20^. The scatter plots of the sample distributions and the classification accuracies are illustrated in Fig. 1.

### Impact of overlap between classes on model performance

We test the effects of overlap between two classes on the classification accuracies of each model by changing the mean and covariance and maintaining the number of samples at 100. In the experiment setup for this case, we use the fixed set of parameters for normal distribution.

**Figure 2:**
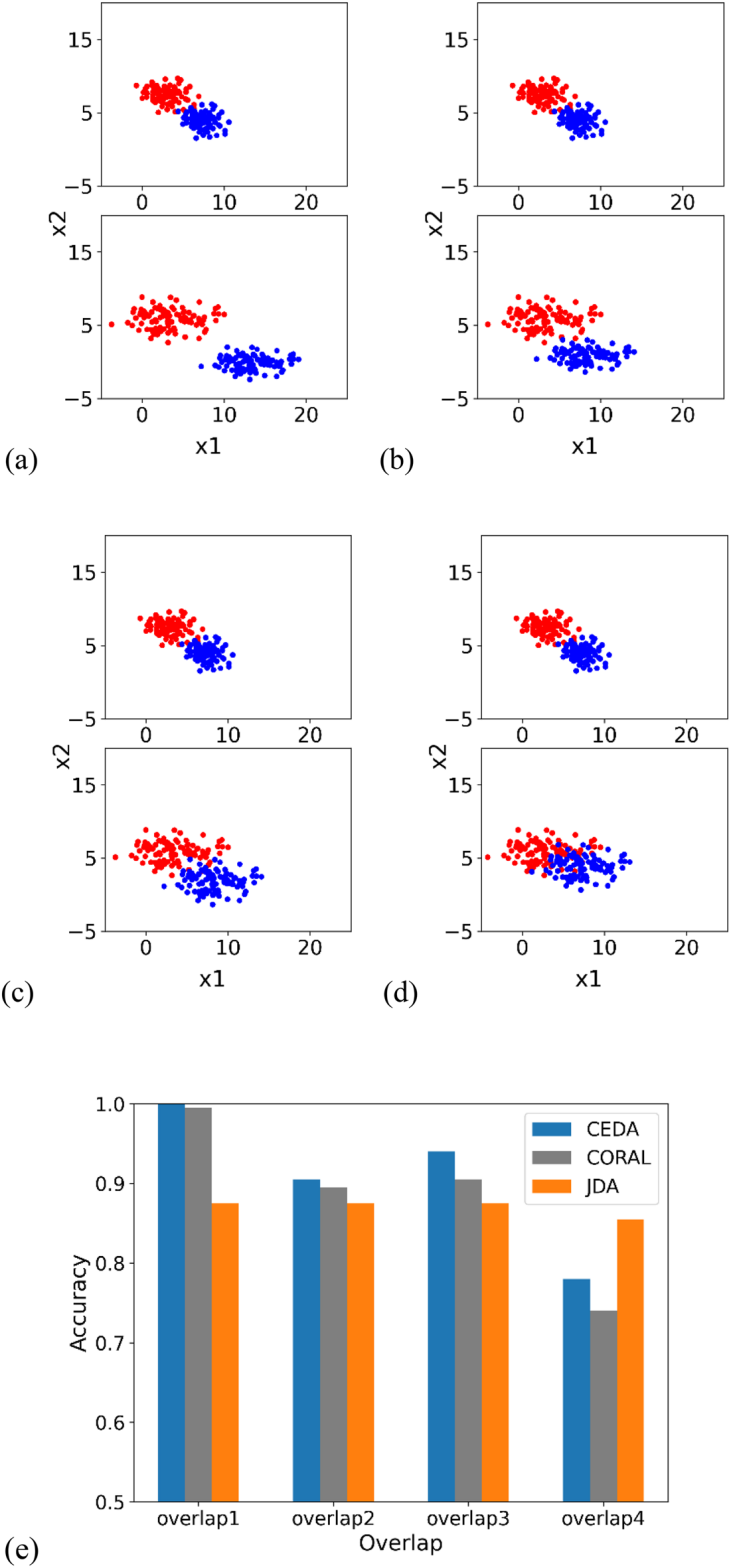
Scatter plots of source samples (upper plot) and target samples (lower plot). Two colors (red and blue) represent two classes. (**a–d**) Depict increasing overlap between classes. (**e**) Denotes classification accuracies at different amounts of overlap.

For source: $${{\varvec{\mu}}}_{1}=\left[\begin{array}{c}2.5\\ 7.5\end{array}\right]$$ and $${{\varvec{\Sigma}}}_{1}=\left[\begin{array}{cc}3& 0\\ 0& 1\end{array}\right]$$, $${{\varvec{\mu}}}_{2}=\left[\begin{array}{c}7\\ 4\end{array}\right]$$ and $${{\varvec{\Sigma}}}_{2}=\left[\begin{array}{cc}2& 0\\ 0& 1\end{array}\right]$$,

For target:

- $${{\varvec{\mu}}}_{1}=\left[\begin{array}{c}3\\ 6\end{array}\right]$$ and $${{\varvec{\Sigma}}}_{1}=\left[\begin{array}{cc}8& 0\\ 0& 2\end{array}\right]$$, $${{\varvec{\mu}}}_{2}=\left[\begin{array}{c}13\\ 0\end{array}\right]$$ and $${{\varvec{\Sigma}}}_{2}=\left[\begin{array}{cc}6& 0\\ 0& 1\end{array}\right]$$,
- $${{\varvec{\mu}}}_{1}=\left[\begin{array}{c}3\\ 6\end{array}\right]$$ and $${{\varvec{\Sigma}}}_{1}=\left[\begin{array}{cc}8& 0\\ 0& 2\end{array}\right]$$, $${{\varvec{\mu}}}_{2}=\left[\begin{array}{c}8\\ 1\end{array}\right]$$ and $${{\varvec{\Sigma}}}_{2}=\left[\begin{array}{cc}6& 0\\ 0& 1\end{array}\right]$$,
- $$ {{\varvec{\mu}}}_{1}=\left[\begin{array}{c}3\\ 6\end{array}\right]$$ and $${{\varvec{\Sigma}}}_{1}=\left[\begin{array}{cc}8& 0\\ 0& 2\end{array}\right]$$, $${{\varvec{\mu}}}_{2}=\left[\begin{array}{c}8\\ 2\end{array}\right]$$ and $${{\varvec{\Sigma}}}_{2}=\left[\begin{array}{cc}6& 0\\ 0& 2\end{array}\right]$$,

(d) $${\boldsymbol{ }\boldsymbol{ }{\varvec{\mu}}}_{1}=\left[\begin{array}{c}2.5\\ 6\end{array}\right]$$ and $${{\varvec{\Sigma}}}_{1}=\left[\begin{array}{cc}8& 0\\ 0& 2\end{array}\right]$$, $${{\varvec{\mu}}}_{2}=\left[\begin{array}{c}7\\ 4\end{array}\right]$$ and $${{\varvec{\Sigma}}}_{2}=\left[\begin{array}{cc}6& 0\\ 0& 2\end{array}\right]$$,

The scatter plots of sample distributions and the classification accuracies are illustrated in Fig. 2.

### Impact of noise on model performance

In this simulation study, the effect of noise on the classification accuracy of each model is tested. The mean vector μ, covariance matrix Σ, and dimension n are generated as described in “Impact of sample size on model performance”. We generate 100 samples for each class, with noise added to each sample.

The noises $\upepsilon$ are sampled from a uniform distribution, ${\mathcal{U}}_{\left[a,b\right]}$

- $$\mathcal{E}\in \left[-\mathrm{1,1}\right]$$
- $$\mathcal{E}\in \left[-\mathrm{2,2}\right]$$
- $$\mathcal{E}\in \left[-\mathrm{3,3}\right]$$
- $$\mathcal{E}\in \left[-\mathrm{4,4}\right]$$

**Figure 3:**
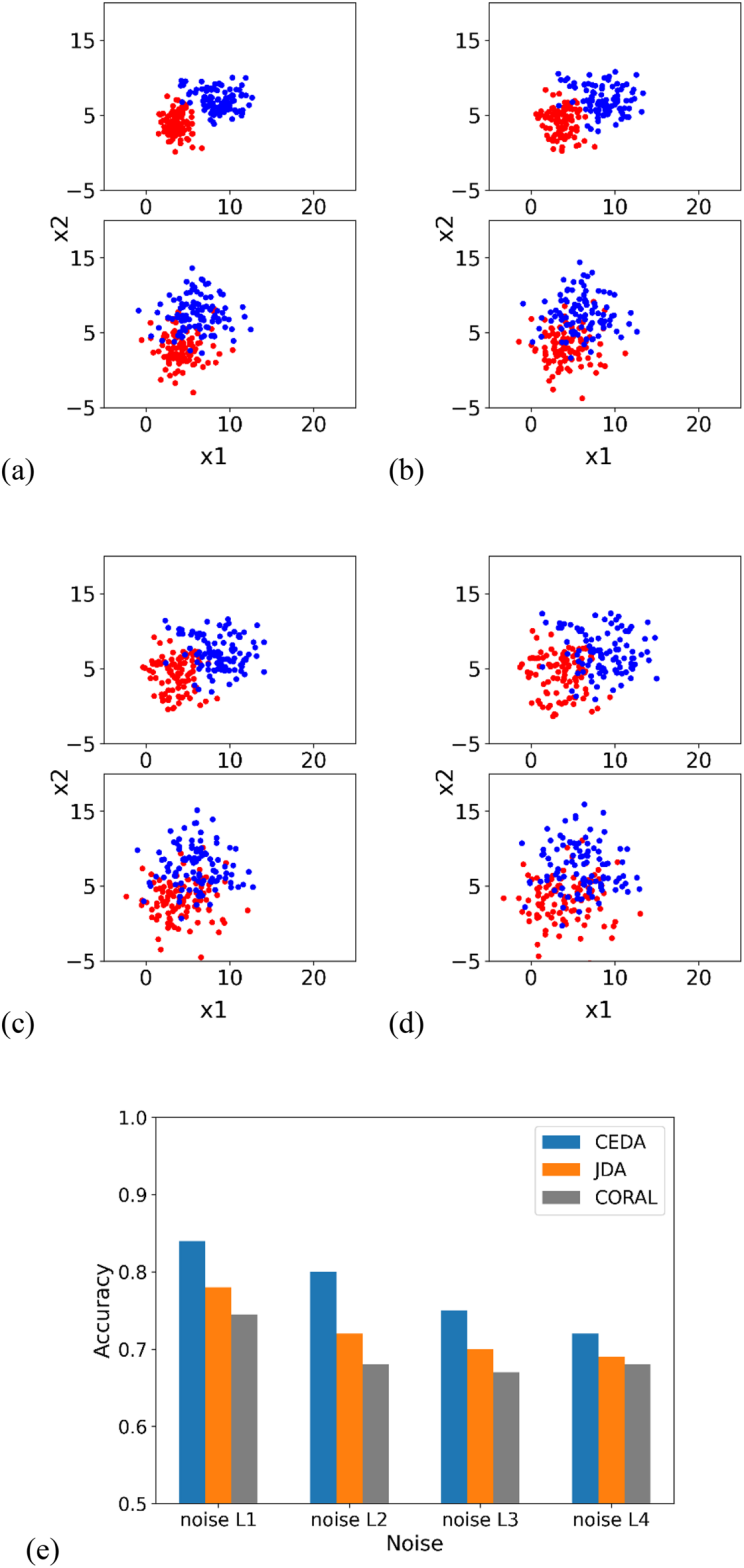
Scatter plots of source samples (upper plot) and target samples (lower plot). We visualize the first and second dimension. Two colors (red and blue) represent two classes. (**a–d**) Illustrate class samples with increasing noise. (**e**) Denotes classification accuracies at different amounts of overlap.

The scatter plot in Fig. 3 illustrates the sample distribution, and the classification results.

### Summary of three experiments

In the three experiments, we tested the robustness of our proposed model by (1) increasing the number of samples in each class, (2) increasing the level of overlap between the two classes, and (3) increasing the noise within each class. The results indicate that our method achieves the highest accuracies compared to JDA and CORAL under the majority of scenarios. The marginal or inferior performance of the proposed method in Figs. 1 and 2 is primarily due to the challenging nature of the datasets under certain conditions, such as significant class overlap. These scenarios are notoriously difficult for most DA methods, and our results reflect these inherent challenges.

### Application in fall risk prediction

In this section, we demonstrate the application of the proposed model to predict fall risk using the dataset obtained from^46^. The human subject experimental procedures followed the principles outlined in the Declaration of Helsinki and gained approval from the Institutional Review Board (IRB) at Virginia Tech (VT), (with assigned protocol codes 11-1088 and study approval date as 10-04-2013). The research took place across four distinct community centers in Northern Virginia—Dale City, Woodbridge, Leesburg, and Manassas. The study employed consistent equipment, specifically Inertial Measurement Units (IMUs), on various days. All research activities were performed in accordance to VT-IRB regulations and guidelines and all participants provided written consent before beginning the study. Participants wear a wearable measurement device and perform a 10-m walking test, from which we extract 50 features related to linear and nonlinear gait parameters for fall risk prediction in two cohorts. The first cohort comprises 171 community-dwelling older adults with known fall information within the last six months. The second cohort consists of 49 osteoporosis patients. All participants underwent the same 10-m walking test following the same guidelines. The challenge is to accurately predict the fall risks of each individual in one group while transferring knowledge from the other group of new patients.

### Data preprocessing

**Figure 4:**
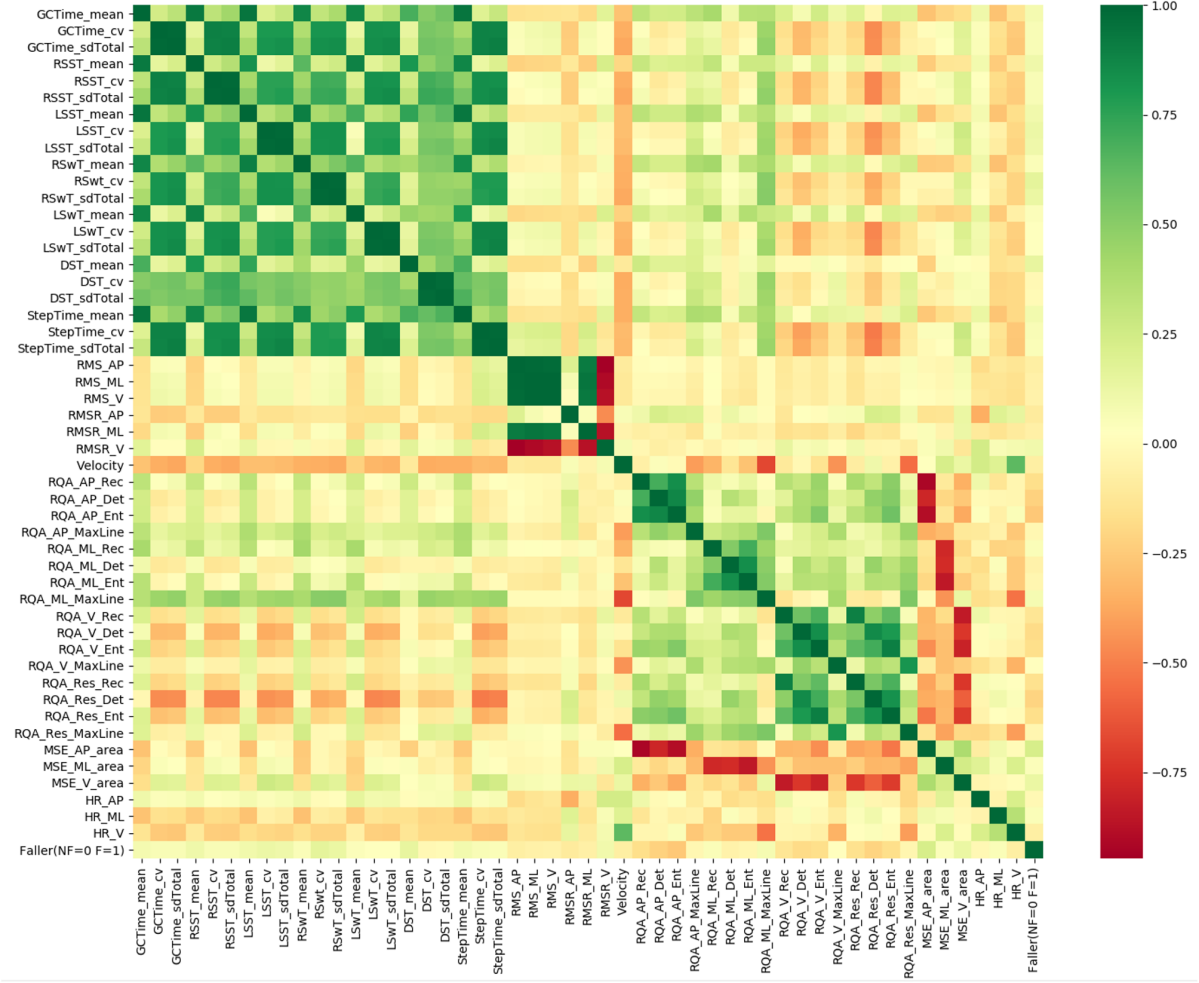
Heatmap of feature correlations.

The dataset comprises 50 features, including 28 linear features (e.g., average step time and walking velocity) and 22 nonlinear features (e.g., anterior–posterior-signal root mean square and vertical-signal maximum line from recurrence quantification analysis). The feature correlations are identical in the two data sources. The feature correlation heatmap (Fig. 4) reveals several highly correlated features. To address potential issues with unstable predictive models and cope with small sample size problems, feature selection and dimension reduction are necessary before applying DA.

### Feature selection dimension reduction

1. Principal components analysis (PCA)^47^
  PCA is a widely used technique for dimension reduction by projecting sample points onto the first few principal components (PCs) to obtain lower-dimensional data while preserving as much variation as possible. In this case study, we calculate 10 PCs from the 28 linear features and 12 PCs from the 22 nonlinear features, and then combine them into 22 PCs. This approach helps minimize the correlation between features within each category of linear and nonlinear features.
2. Filter features based on mutual information^48^
  Mutual information measures the mutual dependence between two variables by quantifying the "amount of information" shared between them. It is equal to zero if and only if two random variables are independent, with higher values indicating a higher dependency. We select the top 10 features from the original set of 50 features based on mutual information.

### Experiment results

**Figure 5:**
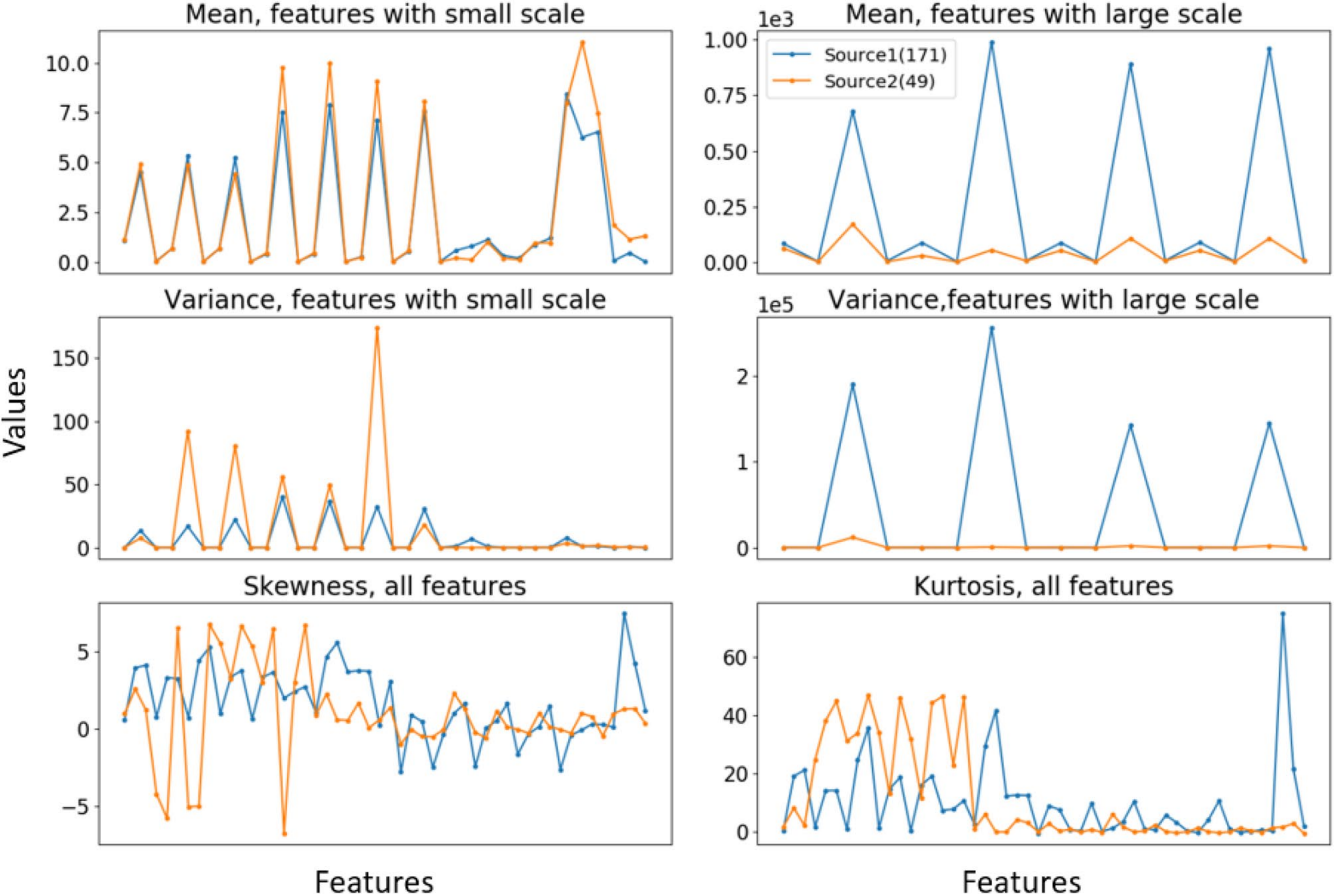
Mean, variance, skewness, and kurtosis of 50 features in two data sources.

The statistics of the two domains also illustrate that the two data sources have different characteristics of features, shown in Fig. 5. Therefore, we must adapt them for better use. Table 1 presents the classification results of directly applying models trained on the source domain to the target domain. We utilized seven classic classification models: support vector machine (SVM)^2^, logistic regression (LR)^49^, decision tree (DT)^50^, k-nearest neighbors (KNN)^51^, random forest (RF)^52^, gradient boosting machine (GBM)^53^ and extreme gradient boost (XGBoost)^54^. To minimize bias caused by a single method, we calculated the average of five classification accuracies.

**Table 1:** Classification accuracies based on source data and accuracies of directly applying models trained on source and target domains.

Classifiers	Classification accuracy based on source		Training on source testing on target	
	Train acc	Test acc	Train acc	Test acc
SVM	0.95	0.69	0.97	0.47
LR	0.64	0.75	0.79	0.66
DT	0.93	0.69	0.91	0.57
KNN	1.00	0.65	1.00	0.67
RF	0.96	0.72	0.96	0.43
GBM	1.00	0.72	0.72	0.55
XGBoost	0.99	0.71	0.72	0.58
Average	0.92	0.70	0.87	0.56

The experiments were conducted as follows: First, we performed a stratified train and test split on the source samples (171 samples) in an 80%:20% proportion. To address the imbalance in the training data, we applied the synthetic minority over-sampling technique (SMOTE)^6^ and random under-sampling technique for resampling the training set. Next, we used cross-validation to tune the optimal parameters in the classifiers. The classification model with the best parameter setting was trained on the training set and used to predict the labels for both the training and testing sets. Subsequently, we applied the model trained on the source dataset to the target samples. We conducted 15 experimental trials with different train-test splits and calculated the average accuracies as the performance measurement. The results showed that the average testing accuracy decreased from 0.7 to 0.56, indicating that directly applying the trained model from the source domain does not yield satisfactory results for the target domain.

In accordance with^19^, we utilize the 1-Nearest Neighbor Classifier (1NN) as the classifier for a fair and straightforward comparison between the proposed method and baseline methods. Since the labeled source and unlabeled target data are sampled from different distributions, tuning parameters using cross-validation is not feasible. Thus, we evaluate all methods by empirically searching the parameter space to find the optimal settings and report the average results for each method. For JDA and CEDA, we search for the number of bases (k) within the range [2, 3, 4, …, 10] and the regularization parameter (λ) from the set {0.01, 0.1, 1, 10, 100}. For GFK, the parameter dimension (d) is used in the range between 1 to half of the feature dimensions, e.g. for 10 features case, d is within^1–5^. CORAL and EasyTL^55^ are parametric-free methods, therefore, no parameter tuning is needed. The experiments are conducted with different data splits five times, and we report the average accuracy along with the standard deviation.

To ensure a fair comparison and avoid data imbalances, we carefully select samples for the dataset cases: dataset 1 (source dataset) to dataset 2 (target dataset) in a ratio of 34:34 to 10:10, and dataset 2 (source dataset) to dataset 1 (target dataset) in a ratio of 14:14 to 25:25. Due to the 1NN classifier's inability to predict classification probabilities, we do not use AUC (area under the curve) for performance measurement. Our approaches consistently outperform JDA and CORAL individually, regardless of the input features. We conduct experiments using five classic machine learning classifiers, applying the same sample separation. In the source dataset, we split the data into training and testing sets for parameter tuning, and then apply the trained model to the target dataset. The testing accuracy is reported along with the standard deviation in Table 2.

**Table 2:** Classification accuracy of two domain shifts on dataset 1 (171 samples) and dataset 2 (49 samples).

Model	Dataset 1 $\to$ Dataset 2			Dataset 2 $\to$ Dataset 1		
	50 features	10 features	22 PCs	50 features	10 features	22 PCs
JDA	0.62 ± 0.11	0.72 ± 0.12	0.64 ± 0.06	0.57 ± 0.09	0.62 ± 0.06	0.61 ± 0.08
CORAL	0.60 ± 0.07	0.67 ± 0.06	0.61 ± 0.08	0.56 ± 0.04	0.56 ± 0.04	0.55 ± 0.06
GFK	0.55 ± 0.07	0.69 ± 0.10	0.58 ± 0.08	0.59 ± 0.04	0.61 ± 0.05	0.51 ± 0.03
EasyTL	0.66 ± 0.06	0.61 ± 0.08	0.50 ± 0.12	0.56 ± 0.09	0.56 ± 0.03	0.42 + 0.06
**CEDA**	**0.64 ± 0.09**	**0.76 ± 0.07**	**0.69 ± 0.07**	**0.59 ± 0.05**	**0.65 ± 0.04**	**0.62 ± 0.09**
SVM	0.49 ± 0.02	0.52 ± 0.05	0.52 ± 0.05	0.50 ± 0.01	0.55 ± 0.03	0.52 ± 0.04
LR	0.49 ± 0.06	0.56 ± 0.08	0.49 ± 0.06	0.50 ± 0.06	0.54 ± 0.02	0.50 ± 0.55
DT	0.57 ± 0.07	0.55 ± 0.05	0.50 ± 0.00	0.54 ± 0.04	0.48 ± 0.04	0.52 ± 0.02
KNN	0.48 ± 0.05	0.56 ± 0.04	0.42 ± 0.08	0.52 ± 0.03	0.58 ± 0.03	0.52 ± 0.03
RF	0.53 ± 0.04	0.55 ± 0.04	0.49 ± 0.06	0.56 ± 0.03	0.53 ± 0.02	0.52 ± 0.03
GBM	0.54 ± 0.02	0.61 ± 0.12	0.50 ± 0.05	0.50 ± 0.05	0.50 ± 0.04	0.50 ± 0.06
XGBoost	0.50 ± 0.03	0.57 ± 0.07	0.52 ± 0.05	0.49 ± 0.02	0.52 ± 0.05	0.50 ± 0.02

Significant values are in bold.

In the real-world case, the target labels are unknown, and therefore, the experiments presented in Table 3 were conducted using 20 random samples (instead of the previously mentioned 10:10 balanced approach) from the target samples as the testing datasets. The ratio of samples from the source dataset to the target dataset is 34:34 to 20. Additionally, we provide the F1 score to assess whether the model overfits the majority class.

**Table 3:** Classification accuracy and F1 score using 10 filtered features.

Dataset 1 $\to$ Dataset 2	10 features	
	Accuracy	F1
JDA	0.73 ± 0.07	0.62 ± 0.12
CORAL	0.67 ± 0.07	0.59 ± 0.09
GFK	0.75 ± 0.10	0.62 ± 0.08
EasyTL	0.58 ± 0.11	0.52 ± 0.13
**CEDA**	**0.81 ± 0.07**	**0.67 ± 0.18**
SVM	0.39 ± 0.18	0.27 ± 0.24
LR	0.47 ± 0.16	0.33 ± 0.18
DT	0.47 ± 0.20	0.39 ± 0.26
KNN	0.54 ± 0.18	0.42 ± 0.21
RF	0.43 ± 0.15	0.48 ± 0.09
GBM	0.51 ± 0.05	0.43 ± 0.06
XGBoost	0.44 ± 0.10	0.36 ± 0.12

Significant values are in bold.

Previously, we demonstrated how we selected 10 features and the feature score of each feature using mutual information. The provided feature scores indicate the contribution of each feature to the DA.

## Conclusion and future work

This paper introduces a novel approach called CEDA for unsupervised domain adaptation (DA). CEDA is designed to align two domains by creating a domain-invariant feature representation. What sets our research apart from existing studies is that we address the challenges of small sample size and imbalanced healthcare data. Our model surpasses competing methods in accurately predicting fall risks for the target domain (new cohort) without relying on labeled data. In our future research, we plan to explore using signals directly instead of extracted features and incorporate a deep learning architecture to further enhance our approach.

## Data Availability

The data supporting this study's findings are available on request from Dr. Thurmon E. Lockhart, [thurmon.lockhart@asu.edu]. The data are not publicly available since data contains information that could compromise the privacy of research participants.
